# ‘Central’ Actions of Corticosteroid Signaling Suggested by Constitutive Knockout of Corticosteroid Receptors in Small Fish

**DOI:** 10.3390/nu11030611

**Published:** 2019-03-13

**Authors:** Tatsuya Sakamoto, Hirotaka Sakamoto

**Affiliations:** Ushimado Marine Institute, Faculty of Science, Okayama University, 130-17, Kashino, Ushimado, Setouchi 701-4303, Japan; hsakamo@okayama-u.ac.jp

**Keywords:** metabolism, behavior, brain, vision, glucocorticoid, mineralocorticoid

## Abstract

This review highlights recent studies of the functional implications of corticosteroids in some important behaviors of model fish, which are also relevant to human nutrition homeostasis. The primary actions of corticosteroids are mediated by glucocorticoid receptor (GR) and mineralocorticoid receptor (MR), which are transcription factors. Zebrafish and medaka models of GR- and MR-knockout are the first constitutive corticosteroid receptor-knockout animals that are viable in adulthood. Similar receptor knockouts in mice are lethal. In this review, we describe the physiological and behavioral changes following disruption of the corticosteroid receptors in these models. The GR null model has peripheral changes in nutrition metabolism that do not occur in a mutant harboring a point mutation in the GR DNA-binding domain. This suggests that these are not “intrinsic” activities of GR. On the other hand, we propose that integration of visual responses and brain behavior by corticosteroid receptors is a possible “intrinsic”/principal function potentially conserved in vertebrates.

## 1. Introduction

Soon after the discovery of the steroid hormone receptor, establishment of its neuroanatomical localization [1,2,3] laid the groundwork for understanding that the brain, similarly to peripheral tissues, is a target organ for steroid hormones [4]. In the brain, receptors for corticosteroids, glucocorticoids (GR), and mineralocorticoids (MR) can act through a classic, genomic mechanism to elicit changes in behavior and physiology, and these receptors can further function at the membrane to activate cytoplasmic signaling pathways [5,6]. Besides the general metabolic functions, this review will focus on integration of visual and brain behavioral responses as a principal function of corticosteroid signaling, based on recent development of the first constitutive knockouts of GR and MR in small fish, since such functions appear to be conserved in vertebrates, including humans [7], and may be important for nutrition homeostasis.

## 2. A Conserved Principal Function of Corticosteroid Signaling Indicated by Receptor-Knockout Small Fish

In this section, we will describe physiological and behavioral (Table 1) changes associated with constitutive knockouts of GR and MR to provide a novel framework in which to explore implications for food intake and nutrition homeostasis.

### 2.1. Glucocortoid Receptor (GR) Knockout

Knockout approaches in mice have been used to study the roles of GR [15]. However, death occurs after a few hours in homozygous GR-knockout mice [16]. Survival to adulthood is observed for heterozygotes or conditional knockouts, and these mice can be used to study the physiological, endocrine, and behavioral details of GR signaling [15,16,17].

#### 2.1.1. Glucocortoid Receptor (GR) DNA Binding Mutant

A zebrafish mutant harboring a point mutation in the GR DNA-binding domain has no transcriptional activity of GR but is adult-viable [11]. This was a key finding; the homozygous mutant fish cannot be distinguished morphologically from wild type in fresh water. This suggests that hyperosomoregulation is similar to that in wild-type zebrafish, although survival is reduced by 70% compared with wild type and immune function is suppressed [8]. However, placement of the mutant fish in an aquarium that is unfamiliar causes them to become immobile and have reduced exploratory behavior, with no habituation to this stress after repeated exposure. Normal behavior can be restored by treatment with fluoxetine, an antidepressant, or ‘visual’ interactions with wild-type fish. These findings suggest an essential link of glucocorticoid signaling, not only to the stress response, but also to regulation of affective disorders [11].

Brain GRs can consistently be considered to be important for regulating hypothalamus-pituitary-adrenal (HPA) axis function independent of pituitary GR activity in mouse models, and the heterozygotic [16] or forebrain-restricted [18] GR knockout also causes depressive behaviors. In contrast, anxiety is reduced or there are no measurable behavioral effects in mice with total neuronal or glial GR knockout [19] and antisense GR knockdown [20]. However, a transgenic mouse with forebrain GR overexpression had increased anxiety and depressive behavior [21]. The inconsistency of the behavioral results in mice may have occurred because manipulations that are tissue-restricted give incomplete or complicated effects [17]. Such mouse behavior is at best correlated moderately with human affective disorder symptoms, but GR can be thought to contribute to the behavior characteristics of anxiety, despair, and learning phenotypes [15].

Visually regulated behavior in the zebrafish mutant described above [11] was abolished by darkness and slowly recovered after light exposure [12]. The mutant had reduced photoresponsiveness of the dark-adapted retina, and delayed readaptation to light. Genes associated with dopaminergic signaling were among several that were dysregulated in the mutant [22,23,24]. These results suggest that glucocorticoids in zebrafish may regulate visual function by controlling the retinal gene network needed for visual adaptation, and this appears to be conserved through vertebrates [25]. This function occurs in addition to the commonly recognized effect of reverse signaling, in which light perception influences glucocorticoid production via functional connections of the retina to the HPA axis [26].

#### 2.1.2. Glucocortoid Receptor (GR) CRISPR (clustered regularly interspaced short palindromic repeats) Mutant

Facchinello and colleagues [8] generated a CRISPR-induced null mutant of the GR gene. This mutant zebrafish can grow, but survival decreases by 50% compared with wild type. Development of the heart and anterior intestine is impaired, and the fish exhibit increased fat deposition, as also seen in Cre recombinase driver transgenic mice with metabolism-associated tissue-specific knockout [17]. In addition, mice with GR knockout in skeletal muscle are protected against muscle wasting caused by elevated endogenous glucocorticoids, which is related to nutritional deprivation [27,28,29]. Disruption of glucocorticoid signaling in osteoblasts also prevented weight gain and altered glucose and insulin sensitivity in a mouse model [30], showing that GR in the musculoskeletal system also regulates the systemic energy supply [31]. However, cross-talk between the related transcriptional regulators, rather than the established transcriptional route for GR, may be needed for at least some changes in glucose metabolism in the zebrafish liver, which is an important regulator of blood glucose, since transcriptional responses of GR-dependent hypoxia-inducible factors that regulate gluconeogenesis/glycogen synthesis are also activated in the GR DNA-binding mutant zebrafish [11]; no direct comparison of the effects of the two GR mutants on glucose metabolism, fat metabolism or depressive behavior had been reported. Additionally, transcriptional activity linked to immune response appears to be hampered worse in the GR CRISPR mutant than in the DNA-binding mutant [8].

The glucocorticoid-mediated hyperactivity to a light stimulus in larvae is also abolished in a zebrafish GR null mutant, in addition to the hypercortisolemic response and the absence of a cortisol stress response [10].

### 2.2. MR Knockout

MR knockout mice experiments have produced several findings [32]. Constitutive MR knockout mice [33] survive only to about postnatal day 10, and have conditions such as increased plasma renin, angiotensin II, aldosterone (pseudohypoaldosteronism), hyperkalemia, and hyponatremia. Rescue is possible by exogenous salt administration [34], but difficulty with salt balance continues, with chronic activation of the renin–angiotensin system. These effects cause difficulty in studying these mice, and MR signaling has mainly been studied using mouse lines with conditionally altered expression or function of MR [32].

**Figure 1:**
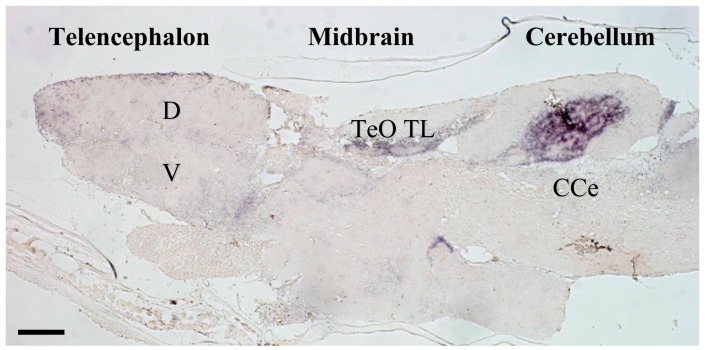
Photomicrograph of mineralocorticoid receptor (MR) in-situ hybridization in medaka brain. MR expression is restricted to a number of important areas that likely correspond to homologous brain regions containing MR in other vertebrates including those of humans [35]. Telencephalic regions exhibiting higher MR expression include the ventral parts of the lateral zone of the dorsal telencephalon (putative fish homologue of the mammalian hippocampus [36]), and commissural and subcommissural nuclei of the telencephalon (V; putative fish homologue to the mammalian amygdala) [36]. In the diencephalon, the hypothalamic preoptic area, inferior lobe of the hypothalamus, and glomerulus complex of the thalamus exhibit MR expression, as do the mesencephalic tegmentum and granular layer of the optic tectum. MR expressed markedly in some regions of the cerebellum. CCe, corpus cerebelli; D, dorsal telencephalic area; TeO, tectum opticum; TL, torus longitudinalis; V, ventral telencephalic area. The detailed expression profiles are described in Sakamoto et al. [13]. Ubiquitously expressed GR in the brains of rodent and fish, as well as MR expression in the rodent brain, have been illustrated elsewhere [15,37,38]. Scale bar: 100 μm.

We recently established a medaka null mutant of the MR gene that grows and adapts to seawater and fresh water [13]. This corresponds to the idea that MR is not involved in body fluid regulation in fish. However, our study of behavioral phenotypes based on the central MR localization (Figure 1) showed these knockout medaka cannot track moving black dots, despite having increased swimming acceleration [13]. Thus, MR is needed for normal motion as the fish responds to visual stimuli, but not for recognition of these stimuli per se. Larval hyperactivity to a light stimulus is also abolished in a zebrafish null mutant of the MR gene, in addition to a delayed but sustained cortisol response to a post-stressor [10].

Medaka with MR have induced GR expression in the eyes and brain, but not in osmoregulatory organs [14]. This shows that glucocorticoid signaling, and not mineralocorticoid signaling, is important in body fluid regulation of the fish [13,39]. GR is activated by cortisol and cortisone, but not 11-deoxycorticosterone, whereas MR is activated by 11-deoxycorticosterone and cortisol, but not cortisone [40]. Therefore, as mostly found in the mammalian brain [41], cortisol is an MR agonist only in tissues without substantial activity of 11β-hydroxysteroid dehydrogenase type 2, which converts cortisol to cortisone [42]. The cellular localization of 11β-hydroxysteroid dehydrogenase in the fish brain is unclear [43], but cortisol-GR might compensate for a lack of cortisol-MR, rather than of 11-deoxycorticosterone-MR. Thus, this GR expression suggests that MR knockout phenotype is due to the loss of 11-deoxycorticosterone-MR.

In neuroendocrine functions of stress, anxiety, and cognition, MR has key roles, as also shown in mouse with conditionally altered expression of MR and in mammals using MR antagonists [15,32,44]. Blockade or knockout of brain MR damages memory and the HPA axis [33,45,46,47,48,49,50,51,52,53], whereas transgenic MR overexpression in forebrain reduces anxiety in mice [54]. Functions of MR mediated by the eyes have not been analyzed by disruption of MR in a mouse model. Cre recombinase driver transgenic mice, which are more tightly regulated, allow temporal induction by doxycycline or tamoxifen, for example, in different life-cycle phases, and they are needed for transgenic studies. These studies may provide more information on the corticosteroid receptor functions.

## 3. Association with Nutrition and Perspectives

Impaired vision-/brain-dependent behavior observed in zebrafish corticosteroid-receptor mutants might cause the defects in behavior of MR-knockout medaka upon exposure to visual stimuli. This mechanism might further regulate corticosteroid-receptor neuroendocrine functions which is well known to be controlled by light/visual perception [55], and the modulation of HPA function may particularly affect food intake and energy homeostasis [15]. A less dissipative metabolism for low stress responsiveness is often associated with fattening [56]. Regardless, our results may indicate a principal function of corticosteroids that is conserved in vertebrates. Corticosteroids may integrate the HPA axis and limbic system with the visual physiology of the sensory periphery, in a coping mechanism for adjustment of the whole organism to rapid changes in environmental conditions, such as visual stimuli. Thus, the fish models described here provide a unique opportunity for identification of corticosteroid-regulated important networks conserved in vertebrates, including humans.

The null mutant of the GR gene has some peripheral changes in metabolism. However, recent evidence demonstrating transcriptional activation of associated GR-dependent genes in the GR DNA-binding mutant suggests that regulation of expression of these genes by GR is not DNA binding–dependent (is not due to “intrinsic” activities of GR) and that such noncanonical, more complex regulation that is widely conserved in human nutrition metabolism requires additional mutational studies [9,57,58]. Targeted genome editing technology permits high-throughput manipulation of corticosteroid receptor-binding sites in transcriptional regulatory regions or protein interaction domains associated with transcriptional regulatory complexes. This technology has been used in several species and may improve knowledge of corticosteroid signaling in human nutrition metabolism [59,60]. Identification and targeting the primary transcripts of corticosteroid receptors will allow determination of the associated regulatory networks. This understanding of signaling mechanisms in human nutrition homeostasis may then lead to new therapies.

The terms “glucocorticoid” and “mineralocorticoid” originate from classical studies in mammals but use of these terms is questionable for all vertebrates. This is based on the essential corticosteroid role in integration of visual responses with brain-dependent behaviors in fish. For example, “glucocorticoid” functions may not be “intrinsic” activities of GR, and mammalian renal mineralocorticoid signaling might have developed during evolution of the loop of Henle, a well-known mammalian target for mineralocorticoids [61]. On the other hand, the essential function of vision-/brain-dependent behavior control that also occurs in mammals is mediated non-genomically as well as genomically [62], which suggests a corticosteroid function that is strongly conserved among vertebrates, including humans.

## Figures and Tables

**Table 1:** Phenotypic consequences including those associated with nutrition dysregulation in constitutive knockouts of glucocortoid receptor (GR) and mineralocorticoid receptor (MR).

Mutant Line	Survival	Morphology	Fat Deposition	Glucose Metabolism	Immune Responses	Osmo-Regulation	HPA Axis	Anxiety	Visual-Regulated Behavior	References
GR Knockout	−	− development of heart and intestine	+	− hypoxia-inducible factor	−	± in fresh water	− negative feedback		−	[8,9,10]
GR DNA Binding Mutant	−	±		± hypoxia-inducible factor	−	± in fresh water	− negative feedback	+	− visual adaptation in retina	[11,12]
MR Knockout	±	±				± in fresh water and seawater	− negative feedback		−	[10,13,14]

Abbreviations: + = increase; − = decrease; ± = no change compared to control fish. Blank columns indicate “not examined”.
